# Medical and Physical Intelligence

**Published:** 1802-02

**Authors:** 


### MEDICAL AND PHYSICAL INTELLIGENCE.

### [ FOREIGN AND DOMESTIC. ]

Dr. SCHMIDTMANN, of Ofnabruck, has given in Hufeland's Journal, an account of the impofition in the Cafe of nearly two years abftinence from all food and drink, which we related in one of the former numbers of this Journal upon the authority of feveral publications that had appeared on this fubject, particularly of a pamphlet written by the above gentleman, in which the whole cafe was ftated, and fome remarks added refpecting the poffibility of fuch a long abftinence, which the author endeavoured to countenance by appealing to the records of fimilar cafes, and by theoretical reafoning, according to which he fancied the patient might have drawn fufficient nourifhment from the elements of the atmofphere, particularly from the oxygen. The fubfequent difcovery, however, will fhow how much the Doctor's learning and fagacity had been mifplaced. Reports were even then fpread of the whole being an impofture, in order to excite pity, and to extort prefents from the people who went to fee this miraculous girl. Though watched by attentive men, fhe had found means to deceive them, till at laft fome young lawyers and phyficians took the pains of guarding her for feveral days, and by their great attention, they fucceeded in difcovering the patient to have excretions of urine as well as of fæces. Though fhe abftained during four days from food and drink, her violent thirft at laft overcame her refolution; and notwithftanding fhe refufed taking any folid food for eight days, at laft fhe was obliged to afk for it. Upon a ftrict examination before the magiftrate it was found, that fhe had, indeed, for a confiderable time, abftained from taking any nourifhment; but inftigated by fome of her acquaintances to take the advantage of the credulity of the public for the purpofe of gaining money and other prefents, fhe had firft conceived the idea of feigning a total abftinence from food and drink, though fhe was all the time fecretly provided with both by her brother, a boy about twelve years, and with the knowledge of her mother. It remains, however, remarkable in this cafe, how the patient had fubfifted upon fuch fcanty provifions as her brother could provide her with, without a proportionable decreafe of flefh and ftrength; and it is curious to obferve, that the auri facra fames could make of a fimple country girl, a moft artful impoftor. For this impofture the girl was committed to bridewell, and the mother and brother, as accomplices, received adequate punifhment; but the father was acquitted, as no proofs could be produced of his having fhared in the impofition.

---

Mr.

Mr. Zurine, of Geneva, Associate of the National Institute, has presented some curious remarks on a small insect, known under the name of water-flea (*monoculus pulex*, L.) which sometimes resides in the greatest abundance in standing water, and has frequently given rise to the rumours of bloody rain, because the great quantity of eggs which it lays in the spring, imparts a bloody colour to the water. Although the best naturalists had made this small animal the object of their researches, such as *Swammerdam, de Geer, Schæffer*, &c. yet Mr. *Zurine* discovered in this small insect very remarkable properties in its structure, oeconomy, manner of coupling, &c. which had escaped the notice of those literati. The most remarkable observation in these animals is, that the females, when once sufficiently impregnated, communicate their faculty of propagation to six successive generations, a phenomenon which has also been observed by *Bonnet*, in the family of Plant-louses, (*aphis*, L.)

---

Dr. Batty will commence his Course of Lectures on the Theory and Practice of Midwifery, and on the Diseases of Women and Children, on Monday the 8th of February, at eleven o'clock in the forenoon. Further particulars may be known at his house in Great Marlborough Street.

*Leadenhall Street, Jan.* 28, 1802.

A report having been circulated, that the Lectures of Dr. Pole and Mr. Wagstaffe on the Theory and Practice of Midwifery and the Diseases of Women and Children, would be discontinued; they take this opportunity of declaring such report to be unfounded; that they commenced another Course on the 20th instant, and will continue to lecture at the usual seasons, of which public notice will be given.

Mr. Macartney will begin his Course of Lectures on Comparative Anatomy and Physiology, early in this month, at the Medical Theatre, St. Bartholomew's Hospital. The Lectures will be given three times a week, and will terminate the end of April.

Mr. Thomas will commence his Spring Course of Lectures on Surgery, on Monday the 1st of February, at seven o'clock in the evening. Particulars may be known at his house in Leicester Square, or at the Anatomical Theatre, Great Windmill Street.

